# Tantalum Interconnect Metallization for Thin-Film Neural Interface Devices

**DOI:** 10.3390/mi17030334

**Published:** 2026-03-10

**Authors:** Justin R. Abbott, Yupeng Wu, Zachariah M. Campanini, Alexandra Joshi-Imre, Felix Deku, Stuart F. Cogan

**Affiliations:** 1 Department of Bioengineering, The University of Texas at Dallas, Richardson, TX 75080, USA; 2 Department of Materials Science and Engineering, The University of Texas at Dallas, Richardson, TX 75080, USA; 3 Department of Bioengineering, Phil and Penny Knight Campus for Accelerating Scientific Impact, University of Oregon, Eugene, OR 97403, USA; fdeku@uoregon.edu; 4 Office of Research and Innovation, The University of Texas at Dallas, Richardson, TX 75080, USA; alexandra.joshi-imre@utdallas.edu

**Keywords:** tantalum, metallization, neural interfaces, thin films

## Abstract

Neural interfaces created using thin-film fabrication rely primarily on conductive metal traces for electrical interconnects. Here, we explore the use of tantalum (Ta) metal interconnects as a replacement for noble-metal interconnects such as Au, Pt or Ir. Ta has been investigated previously for interconnect metallization in flexible silicon ribbon cables, but the structure and properties of tantalum for neural device metallization have not been extensively reported. In the present work, Ta metal was sputter-deposited onto amorphous silicon carbide (a-SiC), with and without a base titanium (Ti) adhesion layer, and investigated as interconnect metallization. In the absence of a Ti adhesion layer, resistivity measurements revealed a factor of six difference between Ta resistivity depending on the presence of the Ti base layer, with direct deposition on a-SiC nucleating high resistivity β-Ta (ρ = 197 ± 31 µΩ·cm, mean ± standard deviation) and Ta deposited on Ti nucleating low resistivity α-Ta (ρ = 35 ± 6 µΩ·cm). X-ray diffraction confirmed the existence of the two crystal structures. Ta feature sizes of 2 µm were created using photolithography and reactive ion etching (RIE). Finally, planar microelectrode array test structures using α-Ta and Au trace metallization with low-impedance ruthenium oxide (RuO_x_) electrodes were fabricated and investigated by cyclic voltammetry (CV) and current pulsing in saline. These devices underwent 500 CV cycles between −0.6 and +0.6 V without evidence of degradation. In response to charge-balanced, biphasic current pulses at 4 nC/phase, a 21 mV increase in access voltage was observed with α-Ta metallization compared to Au. These results warrant further investigation of Ta as thin-film metallization interconnects for neural interface devices.

## 1. Introduction

Thin metal films are used extensively as electrical interconnects in multielectrode arrays (MEAs) employed in neural interface devices. These interconnects are generally metal traces that provide electrical connections from recording and stimulating electrode sites, placed within the cortex or peripheral nerve, to connectors outside the body or to implanted wireless interfaces. Currently, most thin-film interconnects use Au or Pt metals or non-metals such as heavily doped crystalline Si, polysilicon, and crystalline SiC [1,2,3,4,5,6,7,8,9,10,11]. These materials have the benefit of being readily deposited by metal evaporation or magnetron sputtering, or, in the case of crystalline materials like silicon and silicon carbide (SiC), formed from the substrate with which the device is made. The conductive interconnect traces of MEAs are typically compatible with thin-film photolithographic processes and can be patterned to create geometries suitable for a broad range of applications.

Tantalum (Ta) deposited by magnetron sputtering is a promising material for interconnect metallization due to its low resistivity and high corrosion resistance [12,13,14,15]. In biomedical devices, porous Ta has been used in bone and dental implants due to corrosion resistance and good osteointegration [16,17]. Ta is also cyto-compatible, making it promising for use in cellular environments [18,19]. Ta exists in one of two crystallographic forms: α-Ta with a body-centered cubic (bcc) structure and β-Ta with a tetragonal structure [20,21,22]. It has been reported that α-Ta has a lower resistivity (ρ = 20–40 µΩ·cm) by a factor of about six compared to β-Ta (ρ = 150–250 µΩ·cm) [23,24,25]. The crystallographic phase of Ta obtained by magnetron sputtering is dependent on the chemical state of the substrate surface, particularly the presence of oxygen or hydroxide species on the surface [21]. Ta is also compatible with commonly used RIE/ICP dry etch processes and lift-off processes, allowing patterning and dry etching of Ta films. Ta has been employed as a conductor in prior neural interface devices, specifically as an interconnect on ribbon cables for chronically implantable arrays [26].

Recently, ultra-thin MEAs fabricated with amorphous silicon carbide (a-SiC) have shown promise in their ability to record neural activity in the rat motor cortex [27]. The devices in that study employed a Ti-Au-Ti metallization layer deposited via electron beam evaporation. Here, we report the deposition of Ta films onto a-SiC, both directly and using a Ti adhesion layer. We investigated film properties, such as resistivity, crystallographic phase, and film stress. We report that different phases of Ta can be promoted using Ti as a priming layer. We also explored reactive ion etching of Ta with feature sizes as small as 2 µm. Finally, we examined the electrochemical properties of Ta metal in saline using a-SiC test structures coated with sputtered ruthenium oxide (RuO_x_) as a low-impedance electrode coating.

## 2. Materials and Methods

### 2.1. Thin-Film Deposition

Test structures were fabricated on 100 mm diameter, 525 µm thick Si test wafers (Silicon Valley Microelectronics, Inc., Santa Clara, CA, USA). The wafers were initially coated with a 2 µm layer of a-SiC deposited via plasma-enhanced chemical vapor deposition (PECVD) to serve as an isolation layer and substrate for the metallization. The PECVD a-SiC was deposited using a SiH_4_/CH_4_/Ar gas mixture at 133 Pa pressure and substrate temperature of 350 °C, as described previously [28]. All metals were deposited by DC sputtering in an AJA 2200 System (AJA International Inc., Scituate, MA, USA). Metal depositions used a sputtering pressure of 0.53 Pa with Ar at a 50 sccm gas flow, 200 W DC power, and 5 cm diameter targets unless otherwise noted. Nine wafers were coated with Ta (thickness: 314 ± 53 nm (mean ± standard deviation), deposition rate: 11.8 ± 1.6 nm/min) over the a-SiC; seven wafers were coated with Ti (thickness: 49 ± 2 nm, deposition rate: 6.1 ± 0.3 nm/min) followed by a 25 min deposition of Ta (thickness: 264 ± 25 nm, deposition rate: 10.6 ± 0.5 nm/min) without venting the chamber; and three wafers were coated with Ti alone (thickness: 298 ± 9 nm, deposition rate: 6 ± 0.5 nm/min). An additional three wafers of Au-Ti (Ti thickness: 51.1 ± 2 nm, deposition rate: 6.1 ± 0.4 nm/min; Au thickness: 252 ± 5.5 nm, deposition rate: 12.5 ± 0.2 nm/min) were deposited to serve as a high-conductivity comparison, typical of that used in current thin-film a-SiC MEAs [28]. Au was deposited at a power of 100 W. All sputtering targets were parallel to the substrates. In this sputtering chamber, samples are loaded onto a large rotating plate beneath the sputtering sources. Adhesion of the metallization to the a-SiC substrate was assessed qualitatively using a tape-pull test.

### 2.2. Film Characterization

Metal film thickness was measured with a Veeco Dektak VIII Profilometer (Bruker Corporation, Billerica, MA, USA) from metallization deposited onto glass slides included in each deposition. For samples that had a Ti base layer, a base layer thickness of approximately 50 nm was later confirmed via SEM cross-sectional measurements. Sheet resistance was measured using an Alessi 4-point probe system (FormFactor, Inc., Livermore, CA, USA). Resistivity was determined by multiplying the measured resistance by 4.532 (a geometric factor for the four-point co-linear probe measurements) and the measured film thickness. Resistivity is reported in units of µΩ·cm. The crystallographic phase of Ta was determined by grazing incidence X-ray diffraction (GIXRD) using a Rigaku SmartLab high-resolution XRD system (Rigaku America, The Woodlands, TX, USA) with Cu-Kα radiation (wavelength = 0.154 nm) in the range 2θ = 30–100° scanned at a rate of 0.04°/s with a 0.5° incident angle. Based on the thickness of the metal and the use of GIXRD, the incident X-rays do not fully penetrate the top metal layer, and the corresponding XRD spectra are obtained from the outer 30 nm of the top metal layer [29]. The surface morphology and cross-sectional structure of the films were examined by scanning electron microscopy (SEM). Film cross-sections were created from cleaved wafers.

### 2.3. Thermal Annealing

We investigated the effects on Ta metal layers of process temperatures typically associated with a-SiC neural interface device fabrication [28]. Additional Ta alone and Ta-Ti bilayer samples (n = 3 each) were deposited as described above and not coated with a top layer of a-SiC to allow resistivity measurements of the metal films. To mimic the a-SiC PECVD process, samples were placed in a 133 Pa vacuum at 350 °C for 60 min. In addition, we frequently employed a 400 °C vacuum anneal for stress control during a-SiC MEA fabrication, and, consequently, the Ta and Ta-Ti bilayers were then subjected to a 10 min, 400 °C anneal at atmospheric pressure under N_2_ purge. Film stress and resistance measurements were made following metal deposition and then for each of the thermal processing steps. XRD was performed at the conclusion of the heat treatment steps to identify the Ta phase. To examine the as-deposited stress of the Ta-Ti bilayer and Ta metal layers, stress measurements were made against the 2 µm thick a-SiC on silicon, noting that the annealing process likely alters the residual stress of the a-SiC layer such that the stress change on annealing has a contribution from both the metals and the a-SiC.

### 2.4. Dry Etching

We also investigated photolithographic patterning of Ti-Ta-Ti trilayer (the same as the Ta-Ti bilayer with an additional top Ti adhesion layer for overcoating with a-SiC) films by inductively coupled plasma (ICP) reactive ion etching (RIE) (Plasma-Therm, LLC, St. Petersburg, FL, USA). ICP RIE was performed with 25 sccm SF_6_ and 5 sccm O_2_ as reactive gases at 0.53 Pa process pressure and a power of 1200 W. In our a-SiC MEA devices, the target minimum feature size is 2 µm. We patterned the desired metallization with SPR 220-3 positive photoresist (Dow Inc., Midland, MI, USA), which was processed to achieve the desired 2 µm feature size. Etch rate was measured at 30, 45, 60, and 90 s. Samples where the Ta was over-etched to expose the underlying a-SiC were not included in the etch rate determination. Following etching, the photoresist was stripped, and changes in Ta thickness were measured by SEM to calculate etch rate.

### 2.5. Electrochemical Characterization

To examine how Ta metallization impacts the performance of neural interface devices, we created planar test structures with the same insulator–metal–insulator stack as would be used in a device [27,28]. Metallization interconnect layers of thickness 50/250/50 nm of Ti-Ta-Ti (with similar resistivity to the Ta-Ti bilayer described above) and Ti-Au-Ti were patterned. A 2 µm layer of a-SiC was then deposited over the metallization layers. Access vias through the top layer of a-SiC to the underlying metallization were etched using ICP RIE to create circular electrode sites with a surface area of 2000 µm^2^. A low impedance, high charge-injection capacity electrode coating of sputtered RuO_x_ was deposited onto electrode sites as previously described [30]. The RuO_x_ was 350 nm thick, including a 50 nm Ti adhesion layer, and patterned by lift-off photolithography. There were four RuO_x_ electrodes created for each Ta-based and Au-based metallization.

RuO_x_-coated electrode sites were characterized electrochemically by cyclic voltammetry (CV). CV measurements were made in phosphate-buffered saline (PBS), air-equilibrated at room temperature, using a Gamry 600 Potentiostat (Gamry Instruments Inc., Warminster, PA, USA) in a three-electrode configuration with a large-area Pt counter electrode and Ag|AgCl reference electrode. The stability of 2000 µm^2^ RuO_x_ electrodes sputtered onto the Ta and Au interconnects was assessed by 50 mV/s sweep rate CVs for 500 cycles between +0.6 V and −0.6 V vs. Ag|AgCl, which are conservative water window limits for RuO_x_ chosen from prior work [30,31]. RuO_x_ electrode coatings with Ta and Au metallization layers were also evaluated by symmetric, biphasic current pulsing (20 µA current amplitude, 200 µs pulse width per phase, 500 Hz pulsing frequency, 4 nC/phase, 200 µC/cm^2^). Pulsing was performed using a PlexStim system (Plexon Inc., Dallas, TX, USA) with a Pt wire counter electrode. The corresponding voltage transient response was measured with respect to the Pt counter electrode for each metallization layer. The CV cycling stability tests were performed on three RuO_x_ electrode sites for each underlying metal layer, and voltage transient pulsing tests were measured on the fourth, uncycled electrode site for each metallization type.

## 3. Results

### 3.1. Film Characterization

The electrical resistivities of Ta, Ta-Ti bilayers, Ti adhesion layers alone, and Au-Ti bilayers were calculated using sheet resistance measurements and film thickness. The Au-Ti metallization is the same as that used in previous a-SiC MEAs, except that the top adhesion layer of Ti is omitted in this case [28]. Total film thickness for both single and bilayer films ranged from 280 nm to 310 nm. For films with a Ti base layer, the Ti layer was approximately 50 nm and the top metal layer was 250 nm thick. The calculated resistivities are compared in Table 1. Tantalum films deposited directly onto a-SiC were found to have a resistivity of 196 ± 31 µΩ·cm (mean ± standard deviation, n = 7). Ta films deposited onto a Ti interlayer had a lower resistivity of 35 ± 6 µΩ·cm (n = 7), noting that the resistivity is the average calculated over both the Ti and Ta layers. As expected, Au-Ti on a-SiC had the lowest resistivity, 3.2 ± 0.2 µΩ·cm (n = 3). Ti films of a similar thickness to the Ta films (300 nm) had a resistivity of 107 ± 4 µΩ·cm (n = 3), higher than the Ta-Ti bilayer but lower than Ta alone. The results of a one-way ANOVA using Tukey’s post hoc multiple comparisons test are provided in Table 2. We observed that the Ta-Ti bilayer has a lower resistivity than either Ta or Ti alone. The observed resistivities suggest that there is a difference in properties between Ta films deposited directly onto a-SiC and Ta films deposited onto a Ti interlayer on a-SiC. The Ta, Ta-Ti bilayer, and Au-Ti bilayer all deposited on a-SiC passed tape-pull adhesion tests, with no indication of delamination of either the metal from the a-SiC or the a-SiC from the silicon wafer.

**Figure 1:**
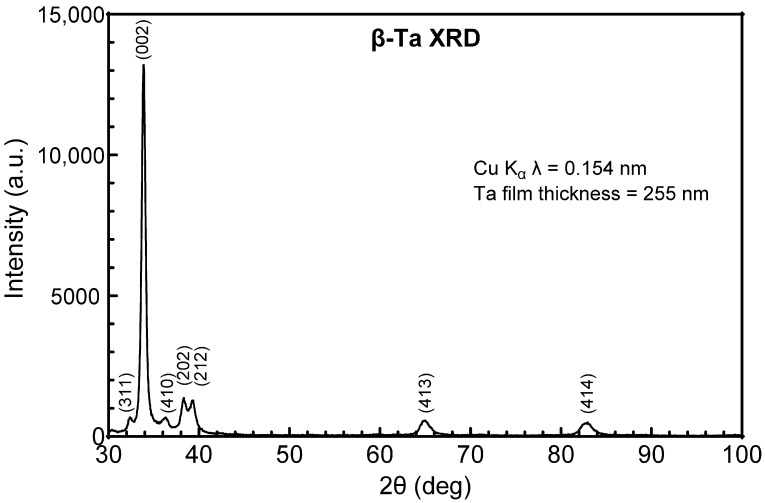
GIXRD pattern of Ta directly deposited onto a-SiC. Identified peaks correspond to bct β-Ta.

**Figure 2:**
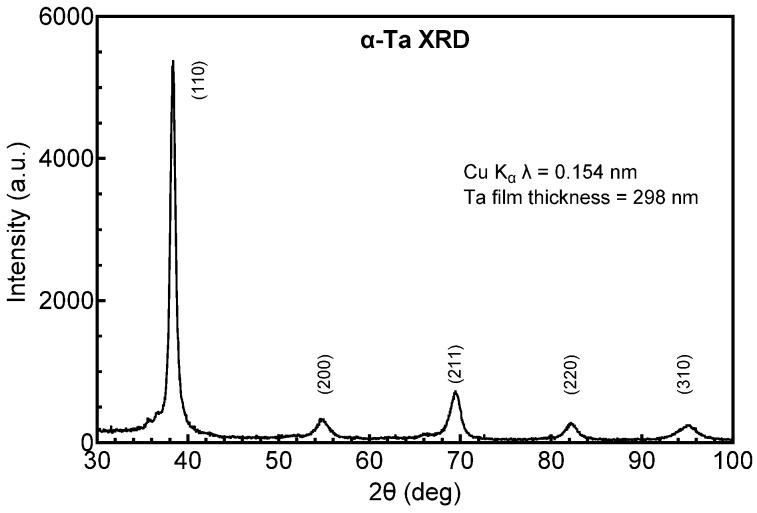
GIXRD pattern of Ta deposited on Ti/a-SiC. The diffraction peaks correspond to bcc α-Ta.

**Figure 3:**
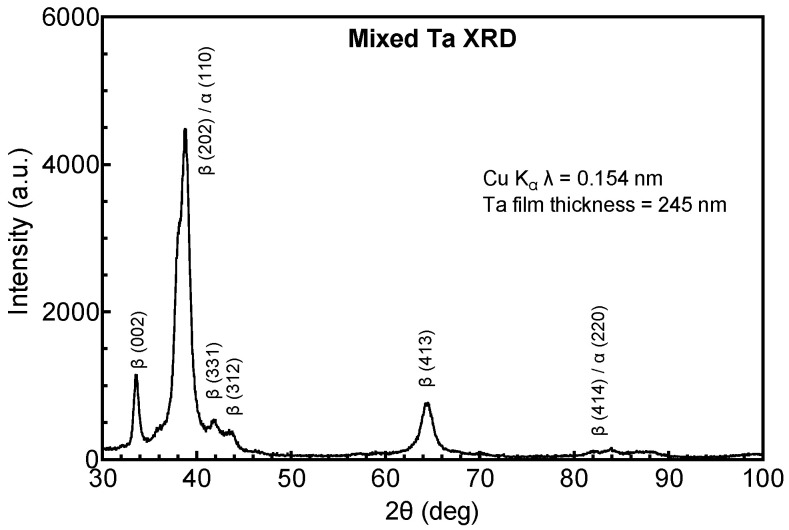
GIXRD of Ta deposited on a Ti interlayer following pump-down to a base vacuum pressure of 6.6 × 10^−4^ Pa. The Ta is predominantly β-phase with a small contribution from α-Ta. The peak labeled as β (202)/α (110) at 2θ = 38° is an overlap of the two reflections.

Possible structural origins of the difference in resistivity of the Ta and Ta-Ti metallization were investigated by GIXRD. Tantalum deposited directly on a-SiC, shown in Figure 1, adopts the body-centered tetragonal (bct) β-phase (JCPDS ref. card 00-025-1280 and JCPDS ref. card 01-070-9756), whereas Ta deposited on a Ti interlayer, shown in Figure 2, adopts the body-centered cubic (bcc) α-phase (JCPDS ref. card 00-004-0788). The initial vacuum base pressure during sputtering was also observed to influence resistivity and crystal structure. Ta-Ti bilayers deposited at a base pressure of 1.3 × 10^−5^ Pa had a low resistivity (25–35 µΩ·cm) with Ta adopting the bcc α-phase. However, bilayers deposited with a comparatively poor base pressure of 6.6 × 10^−4^ Pa had a high resistivity, between 100 and 160 µΩ·cm, and exhibited a mixed-phase structure by GIXRD that was predominately β-phase with a small contribution from α-Ta, as shown in Figure 3. The diffraction peaks were also notably broadened in the mixed-phase films compared with the single-phase spectrum, suggesting a more disordered crystal structure and smaller crystallite size.

**Figure 4:**
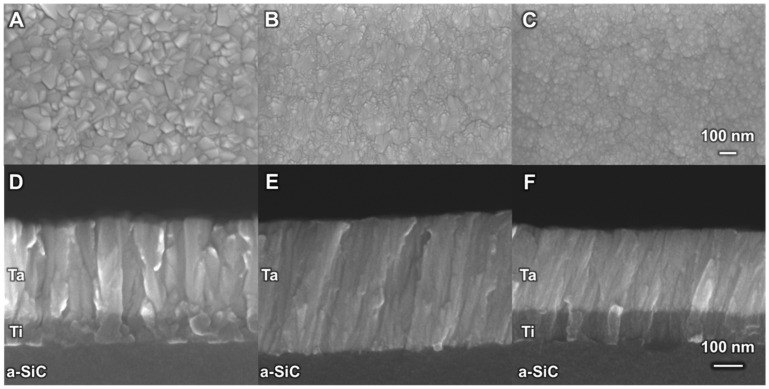
Top-down SEM image of (**A**) α-Ta on Ti, (**B**) β-Ta on a-SiC, and (**C**) mixed-phase Ta on Ti. Cross-sectional profile of (**D**) α-Ta on Ti, (**E**) β-Ta on a-SiC, and (**F**) mixed-phase Ta on a-SiC. Note the differing orientations of film growth for each type of film, with α-Ta having a columnar structure and β-Ta and mixed-phase Ta having a slanted growth morphology. The scale bar in panel (**C**) applies to panels (**A**–**C**), and the scale bar in panel (**F**) applies to panels (**D**–**F**).

Surface and cross-sectional SEM images of the α- and β-phase Ta films and the mixed-phase Ta are shown in Figure 4. Surface SEMs show that α-Ta films (Figure 4A) have a pyramidal-like crystal habit, with a larger crystallite size than β-Ta (Figure 4B) or mixed-phase films (Figure 4C). In cross-section, all three film types had a columnar morphology that was generally more distinct in α-Ta. Interestingly, we observed a slight tilt, 10–15° from perpendicular, in the columnar structure of the β-Ta and mixed-phase films. We note that the Ti interlayer is also more equiaxed in the Ta-Ti bilayer (1.3 × 10^−5^ Pa base pressure) than in the mixed-phase Ta-Ti bilayer (6.6 × 10^−4^ Pa base pressure) films, but the origin of this tilt is uncertain.

**Figure 5:**
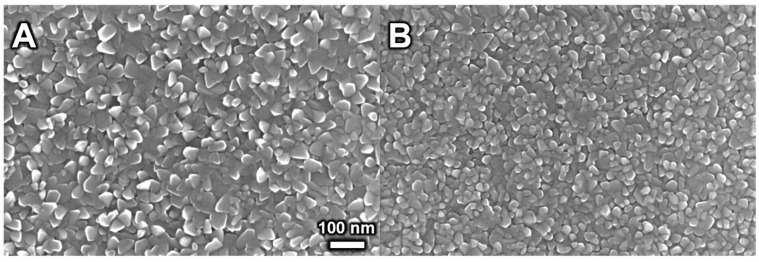
(**A**) Surface SEM of low (1.3 × 10^−5^ Pa) base pressure Ti and (**B**) high (6.6 × 10^−4^ Pa) base pressure Ti films. The lower base pressure (**A**) has a larger crystallite size compared to (**B**). Both films are imaged at the same magnification. Film thickness is 50 nm at either pressure.

**Figure 6:**
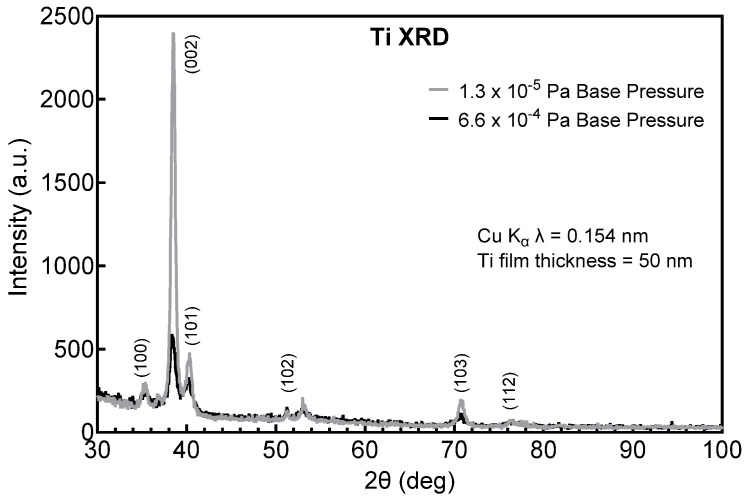
GIXRD pattern of Ti deposited on Ti/a-SiC under (grey) low (1.3 × 10^−5^ Pa) and (black) high (6.6 × 10^−4^ Pa) base pressures. The lower base-pressure films have a higher intensity peak at 2θ = 38°. Both films are 50 nm thick.

As a result of this difference in high base pressure versus low base pressure Ta films, the Ti interlayer was examined as a function of base pressure. The SEM surface morphologies of 50 nm Ti films deposited after pump-down to lower (1.3 × 10^−5^ Pa) and higher (6.6 × 10^−4^ Pa) base pressure are compared in Figure 5. At lower base pressure (Figure 5A), a more pronounced and larger crystal habit is observed than at high base pressure (Figure 5B). GIXRD in Figure 6 shows similar peak positions and relative intensities, although the lower base pressure Ti films, which promote the growth of α-Ta, exhibit notably sharper diffraction peaks, consistent with a larger crystallite size and more defined crystal structure. These differences in the Ti nucleating layer highlight the importance of maintaining a high-vacuum base pressure.

### 3.2. Residual Stress

Sputter deposition of metal films is typically accompanied by significant intrinsic residual stress [32]. Both as-deposited α-Ta-Ti bilayers and β-Ta exhibited intrinsic compressive stresses of −144 ± 10 MPa (n = 3) and −216 ± 56 MPa (n = 3), respectively. Since in our MEA fabrication, the metallization is subjected to temperatures up to 350 °C during PECVD of the outer a-SiC encapsulation, we assessed the effect of a 350 °C vacuum anneal on the residual stress, resistivity, and crystalline phase of the α-Ta-Ti bilayer and β-Ta films. Our fabrication process also employs a 400 °C anneal to balance overall stress in the a-SiC devices. A summary of the resistivity and residual stress after the annealing steps is provided in Table 3. The resistivity and crystalline phase of both α-Ta-Ti bilayers and β-Ta were largely unaffected by thermal annealing at 350 °C and 400 °C, as evidenced by consistency in resistivity and XRD spectra across annealing steps. A modest increase in the compressive stress of the α-Ta-Ti bilayer films with annealing is suggested; however, this increase did not reach significance by repeated measures using one-way ANOVA (*p* = 0.136). In contrast, we observed a large shift in the residual stress of β-Ta from −216 ± 56 MPa to a less compressive value of −10 ± 53 MPa after annealing at 350 °C and a further shift to a tensile residual stress of 132 ± 17 MPa after annealing at 400 °C (*p* = 0.015, one-way ANOVA with a Tukey’s post hoc test). The resistivity of the α-Ta-Ti bilayer was affected by thermal processing, increasing from 26 ± 3 µΩ·cm to 39 ± 6 µΩ·cm after annealing at 400 °C (*p* = 0.012). No significant change in β-Ta resistivity with annealing was observed (*p* = 0.351). The observation that the a-Ta phase is preserved on annealing at 400 °C, at least for 15 min, suggests that the α-Ta phase, and concomitant low resistivity, will be chronically preserved in devices at 37 °C, although additional testing is required to confirm the stability of the α-Ta phase.

Whereas the mechanism underlying the shift to tensile stress in the β-phase has not been definitively identified, a similar observation of increasing tensile stress when heating sputtered or evaporated β-Ta films through 400–430 °C has been reported [33]. Elastic deformation on heating and, at higher temperatures, stress relaxation due to the transformation of β-Ta to α-Ta have been suggested as mechanisms for the stress change. In the present work, GIXRD showed that the film remained in the β-phase when heated to 400 °C. Since the metallization usually occupies a small fraction of the MEA cross-section, less than 5% of the area in a-SiC MEAs [27,28], neither the magnitude nor change in residual stress of the α-Ta-Ti bilayer is expected to have an impact on stress balance in MEAs. We observed a trend of increasing resistivity of the α-Ta-Ti bilayers with the progressive annealing steps (one-way ANOVA, Tukey’s post hoc *p* = 0.012). While this increase is significant, the increased trace resistance is still much lower than that of the β-Ta films. A one-way ANOVA also revealed that there was no significant change in the resistivity of the β-Ta films (*p* = 0.351).

### 3.3. RIE for Patterning Ta Metallization

**Figure 7:**
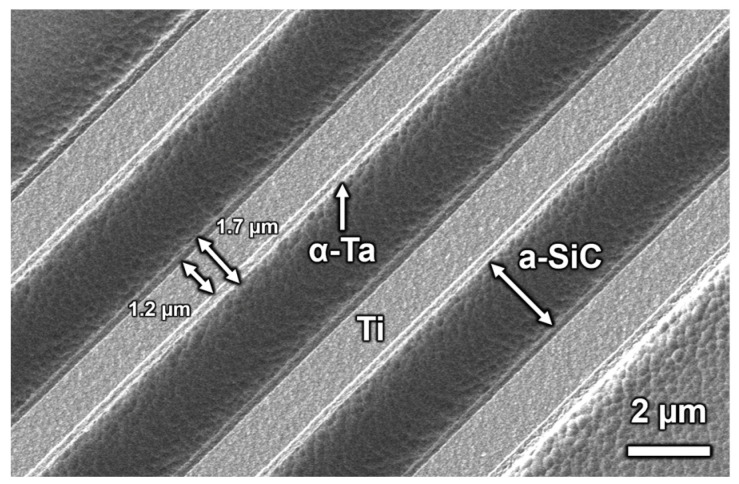
SEM of ICP-RIE patterned traces of Ti-α-Ta-Ti trilayer on a-SiC. These traces were nominally 2 µm in width with 2 µm spacing. The Ta traces are smaller than their intended dimension and are narrower at the top of the trace than at the bottom, with a width varying from 1.2 to 1.7 μm.

Using SF_6_ as the reactive constituent of the plasma, the ICP-RIE etch rate of Ta in Ti- α-Ta-Ti trilayer was approximately 5 nm/s perpendicular to the film surface. Some lateral etching of the traces was observed. As shown in Figure 7, nominally 2 µm wide traces had a width ranging from approximately 1.2 µm on the surface to 1.7 µm after etching a Ti-α-Ta-Ti trilayer (50 nm Ti/250 nm Ta/50 nm Ti) to expose the a-SiC substrate. A lip from the α-Ta and underlying Ti adhesion layer is also evident in Figure 7. Undesirable lateral etching can, to an extent, be avoided by adjusting the width of the photoresist lines and fine-tuning the etching parameters. The 5 nm/s etch rate is slower than that of a-SiC (11 nm/s) under the same ICP-RIE conditions, requiring careful timing of the Ta etch to avoid excessive etching of the underlying a-SiC.

### 3.4. Tantalum Electrochemistry and Current-Pulsing Stability

**Figure 8:**
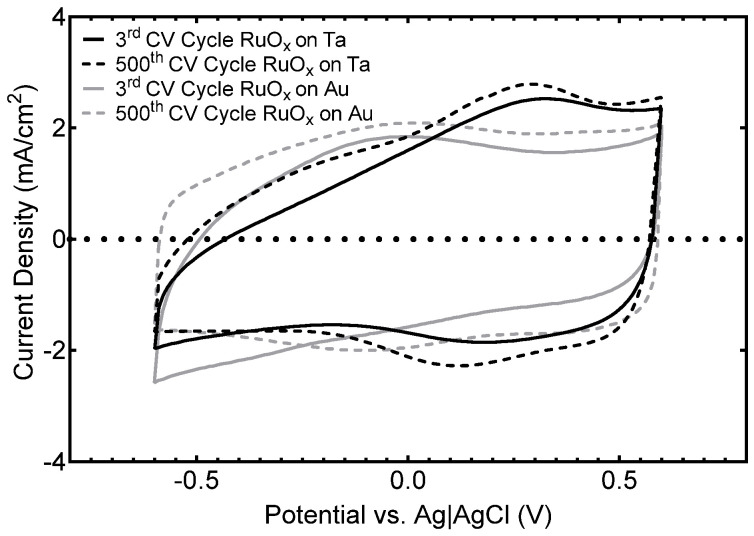
Low-cycle 50 mV/s cyclic voltammetry of RuO_x_ electrodes with α-Ta-Ti and Au interconnects at 3 and 500 CV cycles. The surface area for each RuO_x_ electrode is 2000 µm^2^.

A preliminary assessment of the electrochemical stability of multielectrode arrays employing α-Ta-Ti interconnects was conducted using cyclic voltammetry and short-term current pulsing. Representative cyclic voltammograms of RuO_x_-coated tantalum and gold electrode sites, after 3 and 500 CV cycles, are compared in Figure 8. The RuO_x_ coatings showed expected small changes in CSC_c_ [31] and no evidence of delamination from the underlying Ta or Au metal, noting that a 50 nm Ti adhesion layer was deposited between the Ta or Au and RuO_x_. The RuO_x_ electrodes (250 nm thick, 2000 µm^2^ surface area) deposited on Ta had a cathodal charge storage capacity (CSC_c_) of 39.7 ± 0.8 mC/cm^2^ after the 3rd cycle and 43.3 ± 1.1 mC/cm^2^ after 500 cycles. Similarly, RuO_x_ electrodes deposited on Au contract sites had a CSC_c_ of 39.9 ± 0.9 mC/cm^2^ after the 3rd cycle and 42.3 ± 0.8 mC/cm^2^ after 500 cycles. These values are similar to those reported for other RuO_x_ electrodes on gold electrode contact sites [31]. The increase in the CSC_c_ over time is attributed to a hydration or opening of the pore structure of RuO_x_ during cycling.

**Figure 9:**
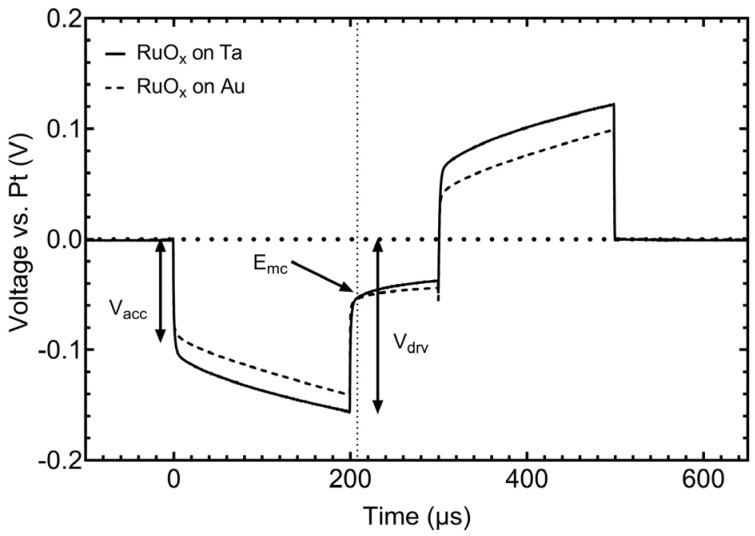
Comparison of the voltage transient response of 250 nm thick RuO_x_ electrodes with α-Ta-Ti and Au-Ti interconnects in response to short-term pulsing of 4 nC/phase. The V_acc_ with the α-Ta-Ti interconnect is larger than that for the Au interconnect by ~20 mV. The RuO_x_ electrode surface area was 2000 µm^2^.

To assess the characteristics of RuO_x_ on α-Ta-Ti for neural stimulation, RuO_x_ electrodes on α-Ta-Ti and Au-Ti underlying metallization were subjected to symmetric, 200 µs (with an interphase delay of 100 µs) rectangular, biphasic current pulsing at 4 nC/phase (200 µC/cm^2^) at a frequency of 500 pulses per second. The voltage transient response was largely the same for both types of metallization, as shown by the comparison in Figure 9. There is a marked difference in the access voltage observed immediately following the application of the current pulse, reflecting the difference in resistivity of the two underlying metal traces. The access voltage for each RuO_x_ electrode was 0.102 V on Ta and 0.082 V on Au, a 0.021 V difference. For both α-Ta-Ti and Au-Ti metallization, the maximum negative potential excursion in response to the cathodal-first pulsing was −52 mV vs. Pt, about 0.8 V positive of the water reduction potential on RuO_x_. The driving voltage (V_drv_) for each electrode was largely influenced by the difference in access resistance. RuO_x_ on α-Ta-Ti had a V_drv_ of 0.156 V, and RuO_x_ on Au-Ti had a V_drv_ of 0.140 V. Lastly, we observed that there was little to no difference in the polarization of the RuO_x_ on either metallization layer, with the electrode on α-Ta-Ti and Au-Ti showing a change of 0.25 mV/µs and 0.27 mV/µs, respectively, during the course of the cathodal phase.

## 4. Discussion

In this work, we assessed the suitability of DC magnetron-sputtered Ta as an alternative to more traditional interconnect metals, such as gold or platinum, in thin-film, multielectrode neural interfaces. Tantalum films deposited by DC magnetron sputtering, under the deposition conditions employed in this study, have moderately compressive residual stress (−130 MPa to −150 MPa for α-Ta and −160 MPa to −270 MPa for β-Ta) and are readily patterned by reactive ion etching, making them potentially useful as interconnect metallization. Reported resistivities of α-Ta and β-Ta range from 20 to 40 µΩ·cm and 150 to 250 µΩ·cm, respectively [20,21,22,34]. We observed similar resistivities, ranging from 28 to 40 µΩ·cm for α-Ta-Ti bilayers and 160 to 220 μΩ·cm for β-Ta. Clearly, obtaining the α-phase is desirable for minimizing trace resistance. We observed that the formation of the α-phase, when sputtering trace metallization onto a-SiC, required the use of a thin sputtered Ti interlayer between a-SiC and Ta. The crystalline phase and orientation of the Ti adhesion layer favored nucleation of α-Ta over β-Ta, although we did not investigate this effect in detail. The importance of the surface of the Ti interlayer in nucleating the α-phase was evident by the occurrence of mixed-phase Ta films on Ti when the base pressure of the sputtering system prior to the deposition of Ta in the Ta-Ti bilayer was not sufficiently low. This observation suggests increased interaction of residual water vapor in the vacuum chamber with the Ti adhesion layer, which leads to nucleation of a mixed-phase Ta film. We could not obtain α-Ta directly on a-SiC, which is consistent with prior studies suggesting β-Ta forms preferentially when depositing onto an amorphous substrate [21]. Thus, a Ti interlayer between the a-SiC and Ta for nucleating the lower resistivity α-Ta is highly desirable.

α-Ta-Ti interconnects provided a stable interface with RuO_x_ electrodes over a limited 500 CV cycle study, with stability similar to that obtained with Au-Ti interconnects. Current pulsing, at charge densities similar to those reported as thresholds for stimulation-induced tissue damage with intracortical microelectrodes [35,36,37], revealed a slight difference in the access resistance between RuO_x_ electrodes on α-Ta-Ti and Au-Ti. However, the electrode on Ta had similar polarization and maximum cathodic potential excursions when compared to Au. These cycling and pulsing results suggest that Ta is a suitable alternative to Au or Pt for metallization from the perspective of neural stimulation charge-injection properties, although long-term chronic stability remains to be established. Additionally, whereas it is possible to dry etch Au by RIE using chlorine-based chemistries, the byproducts of the etch are largely stable and frequently redeposit on the sample and may contaminate the process chamber [38]. Byproducts of Ta chlorine- and fluorine-based dry etching are largely volatile and are purged from the process chamber [39]. These characteristics make α-Ta a possible replacement for Au for metallization interconnects. As noted, lateral etching of the Ta-based metallization using ICP-RIE with the present plasma chemistry undesirably reduces trace line widths, and this presents a challenge when fabricating devices with high interconnect densities. In addition to compensating for lateral etching by photomask design, selection of plasma chemistry and optimization of etching parameters may improve etch profiles and sidewall verticality, as demonstrated by Choi et al. for Cl_2_/Ar gas mixtures [39].

A primary challenge with Ta, compared with noble metals such as Au and Pt, is the higher electrical resistivity of Ta, which inevitably leads to higher trace resistance. The resistivity of α-Ta, including the Ti interlayer, is significantly higher than that of similar thickness Au-Ti interconnect metallization (35 µΩ·cm vs. 3.2 µΩ·cm). Consequently, the higher trace resistance of α-Ta metallization results in more power dissipation during neural stimulation, with the possibility of Joule heating and higher driving voltages for current-controlled stimulation waveforms. Therefore, we estimated the contribution of replacing Au with α-Ta to the overall resistance of an electrode site on a-SiC MEAs being used in intracortical stimulation and recording [27,28]. The electrode sites of these MEAs have a surface area of 200 µm^2^ and are coated with a low impedance film, such as sputtered RuO_x_ or iridium oxide (SIROF) [27,28,40]. Using a resistivity of 35 μΩ.cm, the calculated resistance of an α-Ta trace (with top and bottom Ti adhesion layers), having a 2 µm width and 300 nm bilayer thickness, is 583 Ω/mm of trace length, which compares with 51 Ω/mm for a Au-Ti trace with a 200 nm Au and 50 nm Ti adhesion layer. A similar series resistance was reported by Hetke et al. [26] for sputtered tantalum films on flexible silicon ribbon cables; however, their value of 50 Ω/mm was obtained using 20 µm wide Ta traces, compared with the 2 µm traces used in the present study. For a 2 mm long trace, the increase in resistance of 1 kΩ using the α-Ta metallization is small compared with the typical 1 kHz impedance of 230 kΩ in an inorganic model of interstitial fluid and 1.2 MΩ chronically in rat cortex for 200 µm^2^ SIROF microelectrodes using Au-Ti metallization [28]. Likewise, for neural stimulation with microelectrodes, current magnitudes are typically 10–100 µA, and the additional resistive contribution to the driving voltage would be ≤45 mV for a 2 mm trace length. The contribution of α-Ta trace resistance to the overall driving voltage, which is on the order of 0.5–3 V in animal studies [6,41], is likely inconsequential. Indeed, from the voltage transients in Figure 9, the use of α-Ta-Ti metallization increases V_drv_ by only ~20 mV compared with Au-Ti metallization when pulsing at 20 µA. If the Ta in the trace is β-phase, then the contribution to impedance and driving voltage is increased by a factor of about six. The additional contribution to the driving voltage during current pulsing is significant with β-Ta as the trace metallization and could limit the maximum deliverable current from a stimulator. Additionally, for α-Ta-Ti layers, we estimate that additional power dissipation due to Joule heating during neural stimulation is on the order of 0.18 µW for a 20 nC/phase biphasic pulse with a half-phase pulse width of 200 µs. At a pulse repetition rate of 100 Hz, the average power dissipation during pulsing is 0.2 µW, resulting in a power dissipation of 0.13 watts/cm^2^ of shank surface area for the a-SiC probes used in the present study (2 mm long, cross-section 30 µm × 10 µm). This power dissipation compares with 0.013 watts/cm^2^ for the same MEA geometry with Au-Ti trace metallization. Even simultaneous current pulsing through all 16 α-Ta-Ti interconnects on the a-SiC MEA would increase tissue temperature by only ~0.05 °C, well within reported limits for avoiding thermally induced tissue damage [42]. However, overall device length and implantable depth will be limited by trace resistance. The lengths investigated in the present study provide access to the cerebral cortex, but deeper neural targets, particularly in larger animals, will result in higher resistance and may limit the use of Ta as interconnect metallization in these applications.

Evaluation of Ta in the present study has been limited to a-SiC substrates. The a-SiC is moderately rigid with an elastic modulus of about 76 MPa [43,44] and does not exhibit plastic deformation. However, emerging MEAs that are highly flexible due to the use of ultrathin rigid substrates [28,45] or employ intrinsically flexible substrate materials, such as polyimide or Parylene-C, are actively being developed and used in animal studies [46,47,48]. Additional studies of α-Ta interconnects are necessary to establish stability under cyclic loading and the large flexural displacements that are encountered in these emerging devices. In particular, long-term chronic applications expose interconnects to high-cycle cyclic loads due to tissue motion. The stability of α-Ta interconnects, including both preservation of low electrical resistivity and adhesion to substrate materials, therefore, needs to be established to build confidence for chronic applications, especially for future human use. Likewise, interconnects in devices used for chronic stimulation may be subject to many billions of stimulation pulses with associated repetitive transient thermal effects, which, although, as discussed above, are individually low, do require longer-term pulsing studies of α-Ta interconnects that were not conducted in the present study.

## 5. Conclusions

The results presented here suggest that tantalum thin films deposited by DC magnetron sputtering may be suitable as interconnect metallization on multielectrode arrays used in neural stimulation and recording. With a-SiC arrays, the preferential formation of the low-resistivity α-Ta phase is possible using a Ti interlayer between the a-SiC and Ta, combined with low chamber base pressures prior to sputtering. Based on limited electrochemical cycling, α-Ta is also suitable as an interconnect and electrode substrate for low-impedance electrode coatings such as RuO_x_. To further assess the suitability of Ta as interconnect metallization and replacement for noble metals, additional mechanical durability testing with various substrates, long-term pulsing studies, and assessment of stability in chronic animal preparations are warranted.

## Figures and Tables

**Table 1:** Resistivity of metallization deposited on a-SiC (SD—standard deviation).

Metallization Layer	Mean ± SD Resistivity, ρ (µΩ·cm)	Mean ± SD Sample Thickness (nm)	Number of Samples (n)
Ta	196 ± 31	314 ± 53	7
Ta-Ti	35 ± 6	264 ± 25 (Ta layer)	7
Ti	107 ± 4	298 ± 9	3
Au-Ti	3.2 ± 0.2	252 ± 6 (Au layer)	3

**Table 2:** Resistivity comparisons of metallization deposited on a-SiC (CI—confidence interval).

Metallization Layer	Mean Difference (µΩ·cm)	95% CI	Adjusted *p* Value
Ta vs. Ta-Ti	162	130 to 194	<0.0001
Ta vs. Ti	90	48 to 131	<0.0001
Ta vs. Au-Ti	193	152 to 235	<0.0001
Ta-Ti vs. Ti	−72	−114 to −30	0.007
Ta-Ti vs. Au-Ti	32	−11 to 74	0.021
Ti vs. Au-Ti	104	53 to 154	<0.0001

**Table 3:** Residual stress and resistivity of α-Ta-Ti bilayer and β-Ta thin films.

	α-Ta-Ti Bilayer (n = 3)		β-Ta (n = 3)	
Process Step	Stress (MPa)	Resistivity (µΩ·cm)	Stress (MPa)	Resistivity (µΩ·cm)
As deposited	−144 ± 10	26 ± 3	−216 ± 56	161 ± 10
60 min 350 °C vacuum anneal	−139 ± 4	28 ± 3	−10 ± 53	168 ± 7
15 min 400 °C anneal	−171 ± 15	39 ± 6	132 ± 17	160 ± 4

## Data Availability

The datasets presented in this article are available by request from Stuart F. Cogan, Justin R. Abbott, and Yupeng Wu.

## Abbreviations

The following abbreviations are used in this manuscript:

a-SiC	Amorphous Silicon Carbide
RuO_x_	Sputtered Ruthenium Oxide Film
MEA	Microelectrode Array
SIROF	Sputtered Iridium Oxide Film
CV	Cyclic Voltammetry
